# Association between socioeconomic markers and adult telomere length differs according to sex: Pro-Saúde study

**DOI:** 10.1590/1414-431X202010223

**Published:** 2020-10-07

**Authors:** P. Normando, F.F. Bezerra, B.A. Santana, R.T. Calado, C.B. Santos-Rebouças, E.S. Epel, E. Faerstein

**Affiliations:** 1 Instituto de Nutrição, Universidade do Estado do Rio de Janeiro, Rio de Janeiro, RJ, Brasil; 2 Departamento de Imagens Médicas, Hematologia e Oncologia Clínica, Faculdade de Medicina de Ribeirão Preto, Universidade de São Paulo, Ribeirão Preto, SP, Brasil; 3 Instituto de Biologia Roberto Alcantara Gomes, Universidade do Estado do Rio de Janeiro, Rio de Janeiro, RJ, Brasil; 4 University of California San Francisco, San Francisco, CA, USA; 5 Instituto de Medicina Social, Universidade do Estado do Rio de Janeiro, Rio de Janeiro, RJ, Brasil

**Keywords:** Telomere, Aging, Social determinants of health, Brazil

## Abstract

Understanding the social determinants of telomere length is critical to evaluate the risk of early biological aging. We investigated sex differences on the association between socioeconomic status (SES) and demographic markers and leukocyte telomere length (LTL) in Brazilian adults. This cross-sectional study was conducted in a subsample (women=228; men=200) nested within the Pro-Saúde study, a prospective cohort study of university civil servants in Rio de Janeiro, Brazil (2012-2013). Adjusted multivariate models were used to test the relationship between SES markers (marital status, educational attainment, father's educational attainment, race/skin color, household income, and childhood experience of food deprivation) and LTL. After adjusting for age and potential health-related confounders, lower educational attainment was associated with shorter LTL among men (β=-0.05, 95% confidence interval (CI)=95%CI: -0.10, 0.00, P=0.03). In women, LTL was inversely associated with unmarried status (β=-0.05, 95%CI: -0.09, 0.00, P=0.03), lower father's educational attainment (β=-0.05, 95%CI: -0.13, 0.00, P=0.04), and childhood experience of food deprivation (β=-0.07, 95%CI: -0.13, 0.00, P=0.04). Our findings suggested that the association between SES markers and LTL differs according to sex. SES markers able to induce lifelong stress, reflected in LTL, appeared to be more related to individual factors in men, whereas in women they were family-related.

## Introduction

Socioeconomic status (SES), including education and family income, determines a person's behavior and life conditions, and there is evidence that a lower SES is associated with a higher number and magnitude of chronic stressors (1). It has been proposed that the cumulative biological impact of being chronically exposed to social stressors, such as discrimination (2) and lack of social support (3), can prolong the stimulation of stress response systems, which in turn increases the risk of disease development and accelerates the aging process (4).

Telomeres are DNA repeat sequences (TTAGGG) at the end of chromosomes that reflect cell ability to keep dividing and therefore have been proposed as a biomarker of aging (5). Telomere shortening occurs rapidly during the first few years of life, with a slow progressive shortening being expected to occur throughout life. This process is subjected to the cumulative influence of psychosocial, environmental, and behavioral factors (6) that may accelerate shortening by inducing oxidative stress (i.e., an imbalance between free radicals produced and antioxidants capacity) and inflammation, which cause DNA damage (7).

Several studies have found shorter leukocyte telomere length (LTL) in those with lower SES assessed by different markers. Lower SES based on occupational level was associated with shorter LTL in a cross-sectional study with British female twins (8). Lower educational attainment (9-11) and lower income (12) have also been associated with shorter LTL. However, other studies have found no association between SES and LTL (13-14). Race/ethnicity has also been associated with differences in LTL, with the majority of studies observing longer LTL in blacks compared to whites (10-11). Furthermore, in a population-based sample from USA communities (45-84 years of age), cross-sectional inverse associations between age and telomere length were stronger in black and Hispanics than in whites (15), which the authors proposed could be due to greater social stress.

Brazil is a middle-income country characterized by strong social inequalities, particularly of racial, economic, and/or educational origin, which are known to have health and life expectancy repercussions (16). As gender inequalities are also detected (95th position in the Global Gender Gap Index) (17), we hypothesized that the impact of SES on stress and/or aging, possibly reflected on LTL, may be prone to sex differences. No study has yet explored the association between SES with LTL in Brazil, where studies on telomeres are scarce and usually limited to groups of individuals with specific health conditions (18-20). The aim of the present study was to investigate sex differences on the association between socioeconomic and demographic markers and LTL, in a subsample of a cohort study of civil servants at university campuses in Rio de Janeiro, Brazil.

## Material and Methods

### Study design and participants

This cross-sectional study was nested within the Pro-Saúde study, a prospective cohort study of university civil servants in Rio de Janeiro, Brazil, focusing on the investigation of social determinants of health and health-related behavior (21). Details on the study population and design were previously reported (22). Briefly, four waves of data collection (1999, 2001, 2006, and 2012) have been conducted among 3253 participants. In parallel with wave 4, a subset of 520 participants of wave 1 (baseline) randomly selected within strata of sex, age (less than 50 *vs* 50 years or more), and educational level (less than high school *vs* high school or more) was invited to perform additional interviews that included health-related, nutritional, and body composition data, in addition to blood collection for biochemical and genetic analyses. The study population included a variety of occupations, such as manual workers in charge of maintenance jobs, security personnel, library assistants, health professionals from a university hospital, and other college graduates. Data collection occurred between July 2012 and October 2013.

Participants who either did not have a blood sample (n=8) or who did not respond to socioeconomic or behavioral questions (n=75) were excluded from the analyses, as well as participants self-reported as yellow (n=2) or indigenous (n=7). Thus, a total of 428 participants of both sexes were included in the current analyses.

The study protocol was registered at the Brazilian National Research Ethics System (protocol number: 91642) and approved by the Ethics in Research Committee of the Social Medicine Institute at the Rio de Janeiro State University (CAAE: 04452412.0.0000.5260). All participants provided written informed consent.

### Measurement of leukocyte telomere length (LTL)

LTL was determined in DNA samples extracted from whole blood using a commercial kit (Puregene Blood Kit, Qiagen, Germany). The concentration and quality of the DNA samples were evaluated by spectrophotometry (BioDrop DUO, BioDrop, England). After extraction, DNA samples were stored at -80°C, until the determination of telomere length.

Telomere length was measured by real time quantitative polymerase chain reaction (qPCR) based on the method described by Cawthon, with modifications (23,24). The telomere length for each sample was determined using the telomere to single copy gene ratio (T/S ratio) by the ΔCt method [Ct(telomere)/Ct (single gene)]. The T/S ratio for each sample (x) was normalized to the mean T/S ratio of the reference sample (r) [2-(ΔCtx - ΔCtr) =2-ΔΔCt], which was also used for the standard curve, both as a reference sample and as an inter-assay validation sample. Intra-assay coefficients of variation (CV) for telomere and single gene qPCR analyses were, respectively, less than 2 and 1%. Inter-assay CV was 8.4%.

### Socioeconomic and demographic markers

Sex, race/skin color, educational attainment, and household income were self-reported during the interview. Sex was a dichotomous variable with female treated as the reference. Race/skin color classification was based on official criteria adopted by the Demographic Census conducted by the Brazilian Institute of Geography and Statistics (25) and the participants were self-classified as one of five groups: white, brown, black, yellow, and indigenous. Educational attainment was categorized as high school or less and college education or higher. Father's educational attainment information was categorized as elementary school or illiteracy and high school or higher. Information about monthly household income was collected in ten categories of Brazilian currency units (1 USD=2.05 real): up to R$1000, 1001-1500, 1501-2000, 2001-2500, 2501-3000, 3001-4000, 4001-5000, 5001-6000, 6001-7000, and more than 7000. For income comparisons across households, additional information on number and age of people being dependent on the reported income was used to calculate equivalent per capita household income using the OECD equivalence scale (Organisation for Economic Co-operation and Development) (26). Equivalent household income was categorized into a number of Brazilian monthly minimum wages per capita (<3, 3-6, and >6 minimum wages). Additionally, when used as a continuous variable, equivalent household income was converted to US dollars. Experience of food deprivation (skipping meals due to lack of money - categorized as never, rarely, and sometimes) at 12 years of age was used as a marker of childhood socioeconomic status. Marital status was classified as married or unmarried (single, separated/divorced, or widower).

### Other variables

Individual chronological age, and health and behavioral markers that could affect LTL (6,27,28) were used as covariates, and included: smoking status (categorized as “never smoked” or “ever smoked” if the participant reported being current or former smoker), leisure-time physical activity in the previous two weeks (classified as “yes” or “no”), body mass index [BMI, kg/m^2^ - both the continuous variable and categorized into three BMI strata based on the WHO classification (29): a) underweight or normal, b) overweight, and c) obesity I, II, or III], and self-reported history of medical diagnoses of chronic conditions (“yes” if the participant reported having diabetes, hypertension, hypercholesterolemia, heart attack, angina and/or pulmonary emphysema, or “no”).

### Data analyses

Descriptive statistics were utilized to characterize the study participants. Continuous variables are reported as means±SD (or ±SE) and categorical variables as absolute and relative frequencies. Comparisons between sexes for socioeconomic, demographic, and health and health behavior markers were performed by Student's *t*-test (continuous variables) and by χ^2^ test (categorical variables). Associations between LTL and continuous variables were evaluated by Pearson's correlation analyses.

Mean LTL differences by categories of socioeconomic, demographic, and health and health behavior markers were evaluated by Student's *t*-test (2 categories) or ANOVA (3 or more categories). A univariate general linear model, followed by a least significant difference (LSD) *post hoc* test, was also used to test mean LTL (dependent variable) differences by categories of socioeconomic, demographic, and health and health behavior markers (fixed factors) after adjustment for chronological age (continuous variable), which was included as a covariate.

Multiple linear regression models were used to analyze associations between LTL and SES and demographic markers (model 1, unadjusted), and two other *a priori* defined models were based on the literature (6,27,28): adjusted for age (model 2) and adjusted for age, smoking status, leisure-time physical activity, BMI, and history of chronic disease diagnoses (model 3). For all study samples (combining men and women), models 2 and 3 were also adjusted for sex.

Statistical analyses were performed using SPSS software version 23.0 (SPSS Inc., USA) and P values <0.05 were considered statistically significant.

## Results

The general characteristics of the study population according to sex are presented in Table 1. Mean age was 51.5±7.8 years, varying from 35-79 years among women and 33-77 years among men, and with the majority of individuals of both sexes at the age strata equal to or greater than 50 years old. The distribution by race/skin color was different (P<0.01) by sex although the population was predominantly white for both women and men. No statistically significant difference between sexes was observed for educational attainment, with a higher percentage of individuals having college education or higher (53.1% of women and 56.5% of men). Most of the individuals had fathers with elementary school or illiteracy (64.9% of women and 67.5% of men). Equivalent *per capita* household income was also similar between women and men, with an income less than 3 minimum wages (45.1% of women and 46.5% of men) being more prevalent. Furthermore, there were no differences between women and men in the distribution of childhood food deprivation experience, marital status, physical activity practice, smoking status, BMI categories, and the presence (or not) of chronic disease. Unadjusted mean LTL (T/S ratio) was 0.57±0.16 and was significantly higher in women compared to men (0.59±0.16 and 0.55±0.15, respectively, P=0.01).

**Table 1:** General characteristics of the study population, according to sex: Pro-Saúde study-Rio de Janeiro, Brazil, 2012-2013.

	All (n=428)	Women (n=228)	Men (n=200)	P
	N (%) or mean±SD	N (%) or mean±SD	N (%) or mean±SD	
**Age (years)**				0.05
<50	177 (41.4)	84 (36.8)	93 (46.5)	
≥50	251 (58.6)	144 (63.2)	107 (53.5)	
**Race/skin color**				**<0.01**
Black	82 (19.2)	59 (25.9)	23 (11.5)	
Brown	146 (34.1)	74 (32.5)	72 (36.0)	
White	200 (46.7)	95 (41.6)	105 (52.5)	
**Educational attainment**				0.50
High school or less	194 (45.3)	107 (46.9)	87 (43.5)	
College education or higher	234 (54.7)	121 (53.1)	113 (56.5)	
**Father's educational attainment**				0.60
Elementary school or illiteracy	283 (66.1)	148 (64.9)	135 (67.5)	
High school or higher	145 (33.9)	80 (35.1)	65 (32.5)	
**Equivalent household income (*per capita*, US$)**	1089±687	1090±703	1100±708	0.89
**Equivalent household income (*per capita*, minimum wages)**				0.94
<3 minimum wages	195 (45.6)	102 (45.1)	93 (46.5)	
3-6 minimum wages	172 (40.2)	93 (40.6)	79 (39.4)	
>6 minimum wages	61 (14.2)	33 (14.3)	28 (14.1)	
**Experience of food deprivation (at 12 years old)**				0.13
Never	347 (81.1)	177 (77.6)	170 (85.0)	
Rarely	33 (7.7)	22 (9.7)	11 (5.5)	
Sometimes	48 (11.2)	29 (12.7)	19 (9.5)	
**Marital Status**				0.31
Married	274 (64.2)	151 (66.2)	123 (61.5)	
Unmarried	154 (35.8)	77 (33.8)	77 (38.5)	
**Physical activity practice (in the last two weeks)**				0.08
Yes	175 (40.9)	84 (36.8)	91 (45.5)	
No	253 (59.1)	144 (63.2)	109 (54.5)	
**Smoking status**				0.84
Never smoked	271 (63.3)	143 (62.7)	128 (64.0)	
Already smoked	157 (36.7)	85 (37.3)	72 (36.0)	
**Body mass index (kg/m2)**				0.83
Underweight or normal	122 (28.6)	62 (27.2)	60 (30.0)	
Overweight	172 (40.3)	94 (41.2)	79 (39.5)	
Obesity I, II, or III	133 (31.1)	72 (31.6)	61 (30.5)	
**Diagnosis of chronic disease**				0.37
No	180 (42.1)	91 (39.9)	89 (44.5)	
Yes	248 (57.9)	137 (60.1)	111 (55.5)	
**Leukocyte telomere length (T/S ratio)**	0.57±0.15	0.59±0.16	0.55±0.15	**0.01**

SD: standard deviation. P-values were obtained by χ^2^ test for categorical variables or by Student's *t*-test for continuous variables. Bold type indicates statistical significance.

Age was inversely correlated with LTL (r=-0.11, P=0.02). Equivalent *per capita* household income and BMI were also tested as continuous variables but were not significantly correlated with LTL (Pearson correlation analysis - data not shown).

Mean LTL differences by categories of socioeconomic, demographic, and health behavior markers were explored combining male and female participants (Supplementary Table S1) as well as according to sex (Table 2). In both women and men, no significant difference in age-adjusted LTL was observed by strata of the socioeconomic and demographic markers (Table 2). Among women, LTL was longer in those who did not report physical activity practice in the last two weeks (P=0.04, Table 2), with the opposite being observed among men (P=0.03, Table 2). After adjustment for age, the previously described longer LTL in women (compared to men) was especially evident in those classified as brown race/skin color (P=0.02), with college education or higher (P<0.01), with middle income (equivalent *per capita* household income between 3-6 minimum wages, P<0.01), who were married (P<0.01), who never smoked (P=0.01), who did not report physical activity practice (P<0.01), classified as obese (P<0.01), with history of chronic disease diagnoses (P=0.03), whose father had elementary school or illiteracy (P<0.01), and who reported never experiencing food deprivation at 12 years old (P<0.01) (Table 2).

**Table 2:** Mean values of leukocyte telomere length (LTL) by socioeconomic, demographic, and health and health behavioral markers, according to sex: Pro-Saúde study-Rio de Janeiro, Brazil, 2012-2013.

	Women (n=228)		Men (n=200)	
	LTL age-adjusted (T/S ratio)	P	LTL age-adjusted (T/S ratio)	P
**Race/skin color**				
Black	0.60±0.02		0.55±0.03	
Brown	0.61±0.02		0.56±0.02*	
White	0.58±0.02		0.55±0.01	
**Educational attainment**		0.89		0.07
High school or less	0.60±0.02		0.57±0.02	
College education or higher	0.59±0.02		0.53±0.01*	
**Father's educational attainment**		0.05		0.87
Elementary school or illiteracy	0.61±0.01		0.55±0.01*	
High school or higher	0.56±0.02		0.55±0.02	
**Equivalent household income (*per capita*)**		0.11		0.82
<3 minimum wages	0.57±0.02		0.55±0.02	
3-6 minimum wages	0.61±0.02		0.55±0.02*	
>6 minimum wages	0.60±0.03		0.57±0.03	
**Experience of food deprivation (at 12 years old)**		0.24		0.54
Never	0.60±0.01		0.55±0.01*	
Rarely	0.60±0.03		0.51±0.04	
Sometimes	0.55±0.03		0.57±0.03	
**Marital Status**		0.05		0.93
Married	0.60±0.01		0.55±0.01*	
Unmarried	0.56±0.02		0.55±0.02	
**Physical activity practice (in the last two weeks)**		**0.04**		**0.03**
Yes	0.56±0.02		0.57±0.02	
No	0.61±0.01		0.53±0.01*	
**Smoking status**		0.26		0.51
Never smoked	0.60±0.01		0.56±0.01*	
Already smoked	0.58±0.02		0.54±0.02	
**Body mass index (kg/m2)**		0.29		0.34
Underweight or normal	0.57±0.02		0.55±0.02	
Overweight	0.59±0.02		0.57±0.02	
Obesity I, II, or III	0.61±0.02		0.53±0.02*	
**Diagnosis of chronic disease**		0.50		0.47
No	0.60±0.02		0.54±0.02*	
Yes	0.59±0.01		0.55±0.01	

LTL values are reported as means±SE, (age-adjusted). P values are shown for adjusted univariate general linear model, within each sex. *P<0.05 men *vs* women by univariate general linear model. Bold type indicates statistical significance.

Multiple linear regression models were used to investigate associations between LTL and socioeconomic and demographic markers (model 1), adjusted for age (model 2) or adjusted for age and markers of health and health behaviors (model 3) (Table 3 and Supplementary Table S2). For the study population (combining men and women), models 2 and 3 were also adjusted for sex and all models resulted in no LTL association with socioeconomic and demographic markers (Supplementary Table S2). In women, LTL was inversely associated with unmarried status (Model 3, β=-0.05, 95% confidence interval (CI): -0.09, 0.00, P=0.03, Table 3) and lower father's educational attainment (Model 3, β=-0.05, 95%CI: -0.13, 0.00, P=0.04, Table 3). Moreover, after adjustments, women who reported sometimes experiencing food deprivation in childhood had 0.07 T/S ratio units lower compared to those who did not (95%CI: -0.13, 0.00, P=0.04, Table 3). In men, educational attainment (high school or less) was inversely associated with LTL after adjustment (Model 3, β=-0.05, 95%CI: -0.10, 0.00, P=0.03, Table 3).

**Table 3:** Multivariate associations between leukocyte telomere length (LTL) (T/S Ratio) and socioeconomic, demographic, and health history markers: Pro-Saúde study-Rio de Janeiro, Brazil, 2012-2013.

	Women			Men		
	Model 1	Model 2	Model 3	Model 1	Model 2	Model 3
	β (95%CI)			β (95%CI)		
**Race/skin color**						
White	(ref)	(ref)	(ref)	(ref)	(ref)	(ref)
Black	0.14(-0.04, 0.07)	0.02(-0.03, 0.08)	0.02(-0.03, 0.07)	-0.01(-0.08, 0.06)	0.00(-0.07, 0.07)	0.01(-0.06, 0.08)
Brown	0.03(-0.02, 0.08)	0.03(-0.02, 0.08)	0.03(-0.02, 0.08)	0.00(-0.04, 0.05)	0.00(-0.05, 0.04)	0.01(-0.04, 0.05)
**Educational attainment**						
College education or higher	(ref)	(ref)	(ref)	(ref)	(ref)	(ref)
High school or less	0.00(-0.04, 0.05)	-0.02(-0.06, 0.03)	-0.01(-0.06, 0.04)	-0.03(-0.08, 0.01)	**-0.05(-0.10, 0.00)**	**-0.05(-0.10, 0.00)**
**Father's educational attainment**						
High school or higher	(ref)	(ref)	(ref)	(ref)	(ref)	(ref)
Elementary school or illiteracy	-0.04(-0.08, 0.01)	-0.04(-0.09, 0.00)	**-0.05(-0.09, 0.00)**	0.01(-0.04, 0.05)	0.01(-0.04, 0.05)	0.01(-0.04, 0.05)
**Equivalent household income (***per capita***, US$)**	0.00(0.00, 0.00)	0.00(0.00, 0.00)	0.00(0.00, 0.00)	0.00(0.00, 0.00)	0.00(0.00, 0.00)	0.00(0.00, 0.00)
**Experience of food deprivation at 12 y**						
Never	(ref)	(ref)	(ref)	(ref)	(ref)	(ref)
Rarely	-0.01(-0.08, 0.06)	-0.02(-0.09, 0.05)	-0.02(-0.10, 0.05)	-0.04(-0.13, 0.05)	-0.05(-0.14, 0.04)	-0.05(-0.14, 0.04)
Sometimes	-0.06(-0.10, 0.01)	**-0.07(-0.13, 0.00)**	**-0.07(-0.13, 0.00)**	0.02(-0.06, 0.09)	0.02(-0.05, 0.09)	0.03(-0.04, 0.10)
**Marital Status**						
Married	(ref)	(ref)	(ref)	(ref)	(ref)	(ref)
Unmarried	**-0.05(-0.10, -0.01)**	**-0.05(-0.09, -0.01)**	**-0.05(-0.09, 0.00)**	0.00(-0.04, 0.05)	0.00(-0.04, 0.05)	-0.03(-0.06, 0.00)

CI: confidence interval. Multiple regression models mutually adjusted were used to investigate associations between LTL and socioeconomic, demographic, and health history markers: Model 1: without adjustment; Model 2: adjusted for age (and sex for all group); Model 3: adjusted for age, sex (for all group), marital status, physical activity practice in the last two weeks, smoking status, body mass index, and diagnosis of chronic disease. y: years old. Bold type indicates statistical significance.

## Discussion

In this population of Brazilian adults, we found that the association between SES markers and LTL seemed to differ according to sex. Among male participants, their lower education attainment was the main factor associated with shorter LTL. In contrast, in women, parental SES markers, such as lower father's education and family financial deprivation in childhood, as well as being unmarried appeared to be more relevant in determining shorter LTL. These associations were independent of several biological and behavioral factors recognized to affect LTL, including smoking status, physical activity practice, body mass index, and self-reported chronic disease diagnoses.

Telomere length is usually longer in women than men and this difference does not appear to vary by age group (30). There is also evidence that the LTL shortening process can be influenced by socioeconomic factors (6,10,31). However, whether sex influences the association between LTL and socioeconomic status remains unclear.

Several authors observed a direct association between LTL and SES and demographic markers, such as income (12), married status (32), and higher educational attainment (9,10,33). Regarding the association between LTL and race, previous studies have shown mixed results (15,34). In our racially mixed study population, we did not observe differences in LTL according to race/skin color in either men or women, irrespective of non-whites being categorized in two strata - blacks and browns (i.e., mixed race) - or as a single stratum (data not shown).

Education has been hypothesized to be a more robust indicator of SES throughout the life course (31,33). Given that the effect of SES on telomeres is expected to be the result of many years of exposure to adversity, education has the potential to be more strongly associated to LTL than other SES markers. In fact, a higher educational attainment seems to be associated with longer LTL (31,33,35). Telomere length was directly associated with educational attainment in a study with London-based civil servants aged 53-76 years (33). Similar results were observed in an older (70-79 years) US population, after adjustment for age, sex, and health and behavioral markers (9). A greater number of years of education was also associated with longer LTL in young adults (35 years old approximately) in the Scotland community-based prospective cohort study (35). Furthermore, in a meta-analysis conducted by Robertson and colleagues (31), a higher education level was associated with longer telomeres, whereas other markers of socioeconomic status were not. These studies were all conducted in populations combining men and women and did not report any sex-differences in LTL associations with socioeconomic markers.

In our study, after adjusting for several biological and behavioral factors recognized to affect telomeres, the only marker of SES associated with LTL in men was educational attainment. The relationship was as expected: those men having college education or higher showed longer LTL compared to those having a high school education or less. This is consistent with a more accelerated aging process in less educated men and reinforces potential health consequences derived from socioeconomic inequalities. In a previous study, Pearce and colleagues (11) also observed that educational level was the only LTL predictor in males, while it was not associated in females.

Of note, in the case of women, instead of their own education level, an inverse association was observed between LTL and lower (elementary school or illiteracy) paternal educational attainment. Considering that parents' education is one of the most used indicators of SES during childhood (36), it may be that, in these women, LTL was more sensitive to the influence of SES in the first years of life. This is consistent with life course studies indicating that lower SES (and therefore social stress) earlier in life predicts inflammation and vulnerability to infection in later life (37). Therefore, if the stress caused by lower SES in early life leads to inflammatory damage, paternal educational attainment may capture these effects on LTL more precisely than current socioeconomic circumstances in middle-aged women. Some studies have already observed an association between LTL and parental educational attainment (10,38), however, they did not explore the potential moderating effects of sex.

Strengthening the idea that childhood SES might be more important in determining telomere length in females, we also observed that experiencing food deprivation during childhood was associated with shorter LTL only in women. Failing to have sufficient, safe, and nutritious food characterizes food insecurity that is often associated with low income and poverty (39). There is evidence that food insecurity during childhood has important implications for physical and mental health, as well as social development in the future (39). The stress due to insufficient economic access to food can trigger a cascade of stress hormones and create a metabolic imbalance (40) that may have been captured by LTL. In the US Health and Nutrition Examination Survey (NHANES), Mazidi and colleagues (40) observed that individuals with higher food security status, particularly at a younger age, had longer LTL. Again, sex differences were not explored.

Marital status was an additional SES marker associated with LTL only in women. Longer LTL in married compared to unmarried individuals has been previously observed (32). The benefit of marital relationship to health has been suggested because a partner can provide social support for dealing with life stressors or encouragement to a healthy lifestyle (35). In our study, regarding LTL, it seemed that the benefit of having a partner was limited to women.

Limitations of our study include its cross-sectional nature, limiting causal inferences regarding the influence of socioeconomic and demographic factors on the telomere shortening process. In addition, other differences in LTL by socioeconomic and demographic markers may not have been observed because our study population was a subsample of a cohort study of civil servants at university campuses, having some similar social patterns. We also acknowledge that the relatively small sample size may have limited our conclusions. Nevertheless, the current results associating socioeconomic and demographic markers and LTL add replication and extension.

This is the first study that analyzed sex differences on the associations between LTL and socioeconomic and demographic markers in a middle-income country. Importantly, two-thirds of our participants' fathers had elementary education or were illiterate, a life course situation much less frequent in contemporary high-income countries. Given that Brazil still shows important gender inequalities, it is plausible to consider that the impact of SES on stress and/or aging occurs differently in men and women. Most participants in our study were born during the 1950s and 1960s, in which men used to be the breadwinner, and women were more dependent on relatives' support. Consistently, our study suggests that SES markers able to induce lifelong stress, reflected in LTL, appear to be more individual in men (own education), whereas in women they are family-related (father's education, childhood food deprivation, and marital status).

## Supplementary Material

Click here to view [pdf].
